# Modified Valente Technique for Cauliflower Ear: Outcomes in Children at Two-Year Follow-Up: New Surgical Technique for Cauliflower Ear

**DOI:** 10.1007/s00266-024-03914-5

**Published:** 2024-03-18

**Authors:** Luca D’Ascanio, Eleonora Gostoli, Giampietro Ricci, Pietro De Luca, Gino Latini, Michael J Brenner, Arianna Di Stadio

**Affiliations:** 1 Department of Otolaryngology, Azienda Ospedaliera Ospedali Riuniti Marche Nord, Pesaro-Fano, Italy; 2 Department of Otolaryngology, University of Perugia, Perugia, Italy; 3 3Department of Otolaryngology, Isola Tiberina Hospital - Gemelli Isola, Rome, Italy; 4 Department of Otolaryngology – Head and Neck Surgery, University of Michigan Medical School, Ann Arbor, Michigan, USA; 5 Department GF Ingrassia, Otolaryngology Unit, Catania, Italy

**Keywords:** Ear malformation, Cauliflower ear, Surgery, Reconstruction, Follow-up, Esthetic results

## Abstract

**Background:**

Cauliflower ear deformity, a common sequela of auricular trauma, presents an esthetic and reconstructive challenge. Existing surgical techniques have limitations, including complexity, donor site morbidity, and variable long-term outcomes.

**Materials and Methods:**

In this case series, we present a novel and minimally invasive surgical approach for the correction of cauliflower ear deformity that adapts the Valente otoplasty technique; it combines cartilage debulking with helical rim release and Mustardé mattress stitches to restore ear contour and reduce the risk of recurrence. The procedural steps include bielliptic post-auricular skin and soft tissue incision, release of the cartilaginous spring, removal of excess fibrocartilaginous tissue, cartilage reshaping with suture to restore contour, and tissue redistribution to promote adherence of skin to the cartilage framework.

**Results:**

Outcomes were evaluated in 7 patients (9 ears) with cauliflower ear deformity, assessing surgical duration, complications, patient satisfaction, and esthetic outcomes at two years after surgery. The mean surgical duration per patient was 52 ± 17 minutes, including 2 bilateral procedures. Follow-up at 24 months showed favorable esthetic outcome in all patients with sustained improvements in auricular contour and symmetry with neither loss of the shape nor recurrence of deformity. Patients reported high satisfaction and improved quality of life, with mean Glasgow Children Benefit Questionnaire scores of 99.3 ± 6.3.

**Conclusions:**

This technique thus demonstrated lasting correction of cauliflower ear with favorable cosmetic outcomes, low risk of complications, and high patient satisfaction. Further investigations and longer-term follow-up are warranted to validate the technique's durability and expand its application to older and more diverse patient populations.

**Level of Evidence IV:**

This journal requires that authors assign a level of evidence to each article. For a full description of these evidence-based medicine ratings, please refer to the Table of contents or the online Instructions to Authors www.springer.com/00266.

**Supplementary Information:**

The online version contains supplementary material available at 10.1007/s00266-024-03914-5.

## Introduction

Cauliflower ear most often arises from hematoma after repetitive blunt trauma to the auricle. The pathogenesis involves the accumulation of blood between the cartilage and the perichondrium, resulting in impaired perfusion; the loss of bloody supply and the persistent inflammation cause destruction of the cartilage, with subsequent cartilage fibrosis and ear malformation [1]. The sequelae of cauliflower ear deformity can often be prevented if an auricular hematoma is evacuated swiftly followed by obliteration of dead space with a compressive bolster or sutures [2]. However, if untreated, chondronecrosis occurs and fibrous proliferation ensues, requiring surgical correction to restore contour. The literature on techniques and outcomes of surgery for cauliflower ear is limited [3–6], and complex ear reconstructions entail long operative times, morbidity, and risk of complications [4, 6].

Cauliflower ear can develop at any age after auricular hematoma, and it is of special concern in children, who may be traumatized by stigma associated with the deformity. Due to high rates of recurrence or unfavorable outcomes surgery, treatment of the malformed ear is often not pursued or deferred until adult age [4–7]. Some procedures require prolonged or multistage surgery. Vogelin et al proposed removing the excess fibrotic tissue without cartilage insertion [7] to recreate the malformed helix [8]. This approach afforded satisfactory short-term results; however, relapse was common within one to two years after surgery. We describe long-term results of surgery for cauliflower ear deformity in a cohort of 7 children (two bilateral cases), using a novel procedure adapted from Valente otoplasty technique [9].

## Materials and Methods

The study was conducted in the Otolaryngology department of a tertiary referral center from September 2019 to April 2023, spanning date from first surgical procedure to two-year follow-up. The study was conducted in accordance with the Declaration of Helsinki. All patients were under the age of 18, and parents of all patients signed a written consent that included the authorization to collect, analyze, and report patient data for the research study.

Data were collected on patient characteristics, procedural details, presence of intraoperative or immediately post-surgery complication (e.g., bleeding, infection), objective result of surgery, and patient-reported outcomes. For all patients, data were collected on sociodemographic parameters, comorbidities, and relevant medical history. In addition, the severity of cauliflower malformation was evaluated before surgery using the Manninen assessment [10], which assesses the severity of malformation with scores from 1 (minimal deformity) to 5 (severe malformation). All procedures were performed by a single surgeon (LDA). Duration of surgery was measured from skin incision to placement of the final suture. Intraoperative, immediate postoperative, and late complications were recorded. Photographs taken before and at regular intervals after surgery to assess for esthetic correction over time. The Glasgow Children Benefit Questionnaire (GCBQ) [11] was completed by the parents of all children, and outcomes for all procedures were tracked for a minimum of 2 years.

### Surgical Technique

**Fig. 1:**
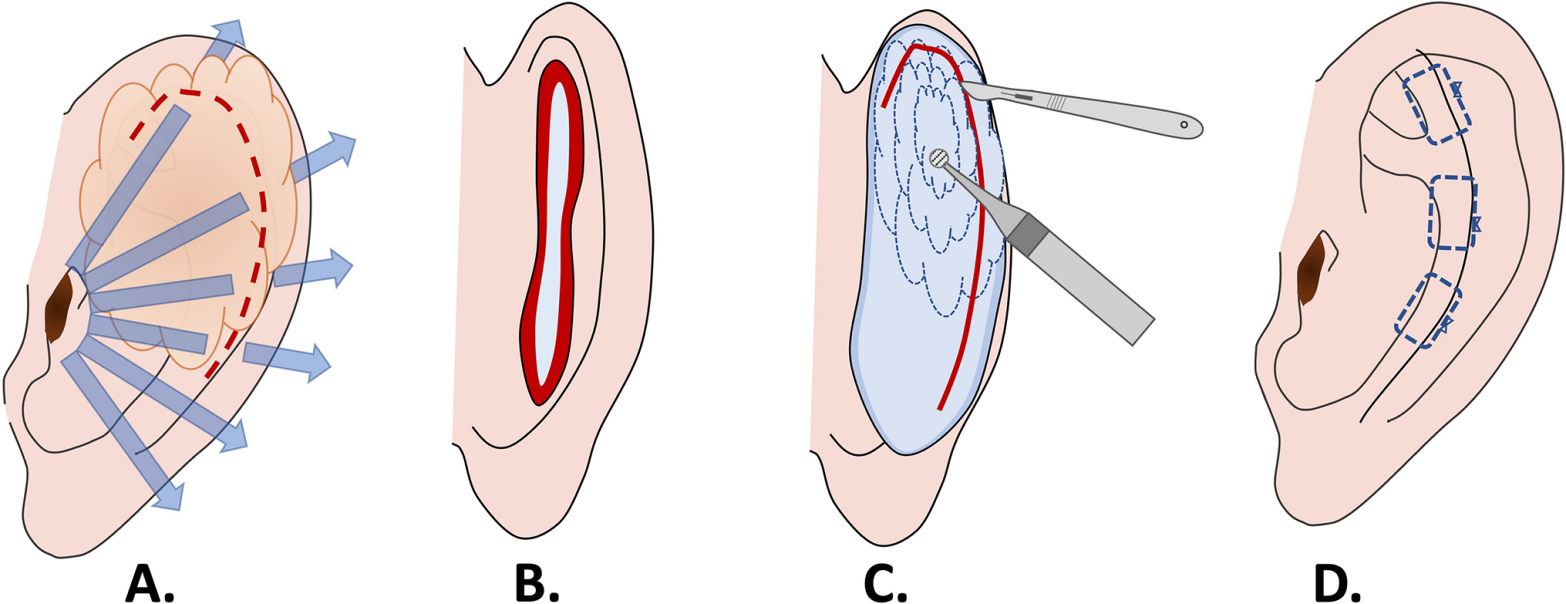
Schematic summarizing steps of the modified Valente technique for cauliflower ear: **A** Cauliflower ear deformity distorts auricular anatomy, and helical release breaks the cartilage spring; **B** bielliptic, peanut-shaped post-auricular skin excision affords access; **C** scalpel excision and diamond burr are used to remove deformed cartilage; **D** contour is restored after mattress suture and redistribution of skin over the cartilage framework

**Fig. 2:**
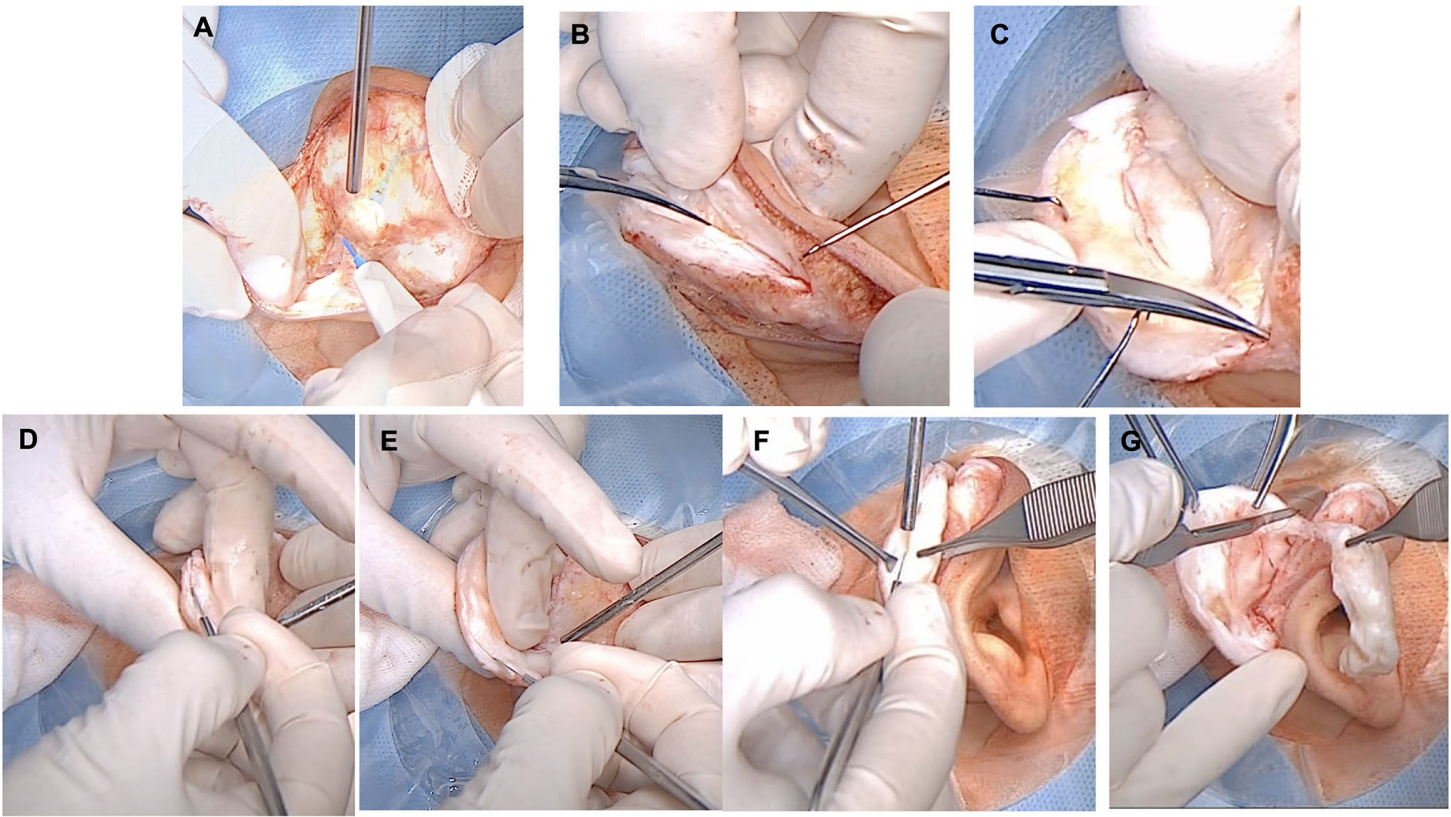
Surgical step 1. **A** Skin is detached from the posterior ear; **B–C** cartilage incision 3–4 mm from the auricle free edge with detachment of the skin from the anterior surface of the pinna; **D, E, F** resection of deformed fibrous tissue from cauliflower ear; **G** thinning and removal of excess tissue

Our approach to correction of cauliflower ear, adapted from Valente otoplasty technique [8], combines cartilage debulking with helical rim release and Mustardé mattress stitches to restore ear contour and reduce the risk of recurrence. The approach is summarized in the schematic image (Fig. 1)***.*** First, the desired position for the new antihelical fold is determined by gently rolling the ear posteriorly. A 27-gauge needle with methylene blue is then used to mark planned suture placement and bielliptic skin excision. After injection of a local anesthesia (2% lidocaine solution with 1: 20000 adrenaline), a #15 scalpel blade is used to excise the skin and subcutaneous tissue down to perichondrium. Next, dissection of the posterior concha is performed just superficial to the perichondrium, proceeding from the mid-conchal bowl posteriorly to the mastoid bone without detaching the cartilage (Fig. 2A). The conchal cartilage is then sharply incised with a #15 scalpel 3–4 mm behind the helical rim, to release the cartilage spring; this incision is followed by scissor dissection to access to the malformed cartilage (Fig. 2B, C). Preserving perichondrium in the posterior portion of the cartilage ensures adequate perfusion and preservation of support.

**Fig. 3:**
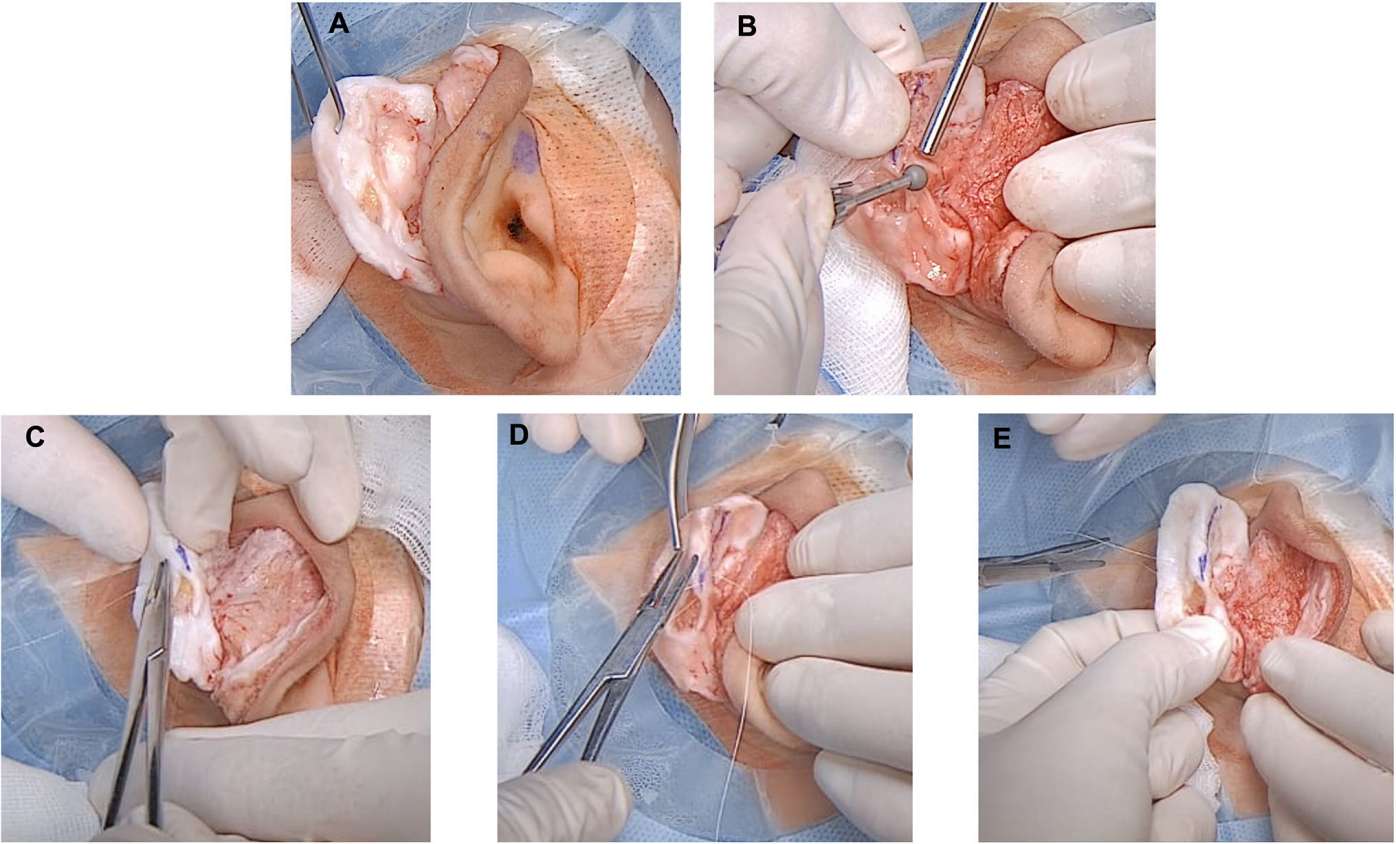
Surgical step 2. **A** Auricular cartilage after removal of the deformed portion; **B** auricular cartilage smoothing by diamond burr; **C–E** use of Monocryl mattress sutures to stabilize the new antihelix

Using a caliper, the volume of fibrotic tissue for removal is determined; normal concha cartilage is around 1 mm thickness [9]. Methylene blue markings are made to determine the extent of tissue removal. Excess tissue is sharply excised in superior to inferior and external to the internal directions (Fig. 2D–G). After removal of excess tissue from the anterior aspect of the ear (Fig. 3A), a 4mm diamond bar or cutting burr is used to increase flexibility of the ear, achieve consistent thickness, and refine contour (Fig. 3B). The cartilage is then folded to recreate the new antihelix, with any excess cartilage in the superior part of the concha removed. The fold is then maintained using mattress sutures [12] that incorporate perichondrium, using resorbable 4-0 Monocryl suture [8] (Fig. 3C–E). The new antihelix is again contoured with the diamond burr, and the skin is redistributed over the cartilage framework and sutured. Gauze with Fitostimoline® (DAMOR spa, Naples, Italy) is placed over the anterior and posterior ear, followed by placement of a compressive bandage [13].

*Statistical analyses.* We performed two-tailed t-test to compare the difference in the GCBQ between patient who had worse (≥3 points) malformation score versus lower at the baseline. Two-tailed t-test was also performed to compare the GCBQ in patients who underwent bilateral versus unilateral surgery. P was considered statistically significant <0.05. All tests were performed by Stata ®.

## Results

**Fig. 4:**
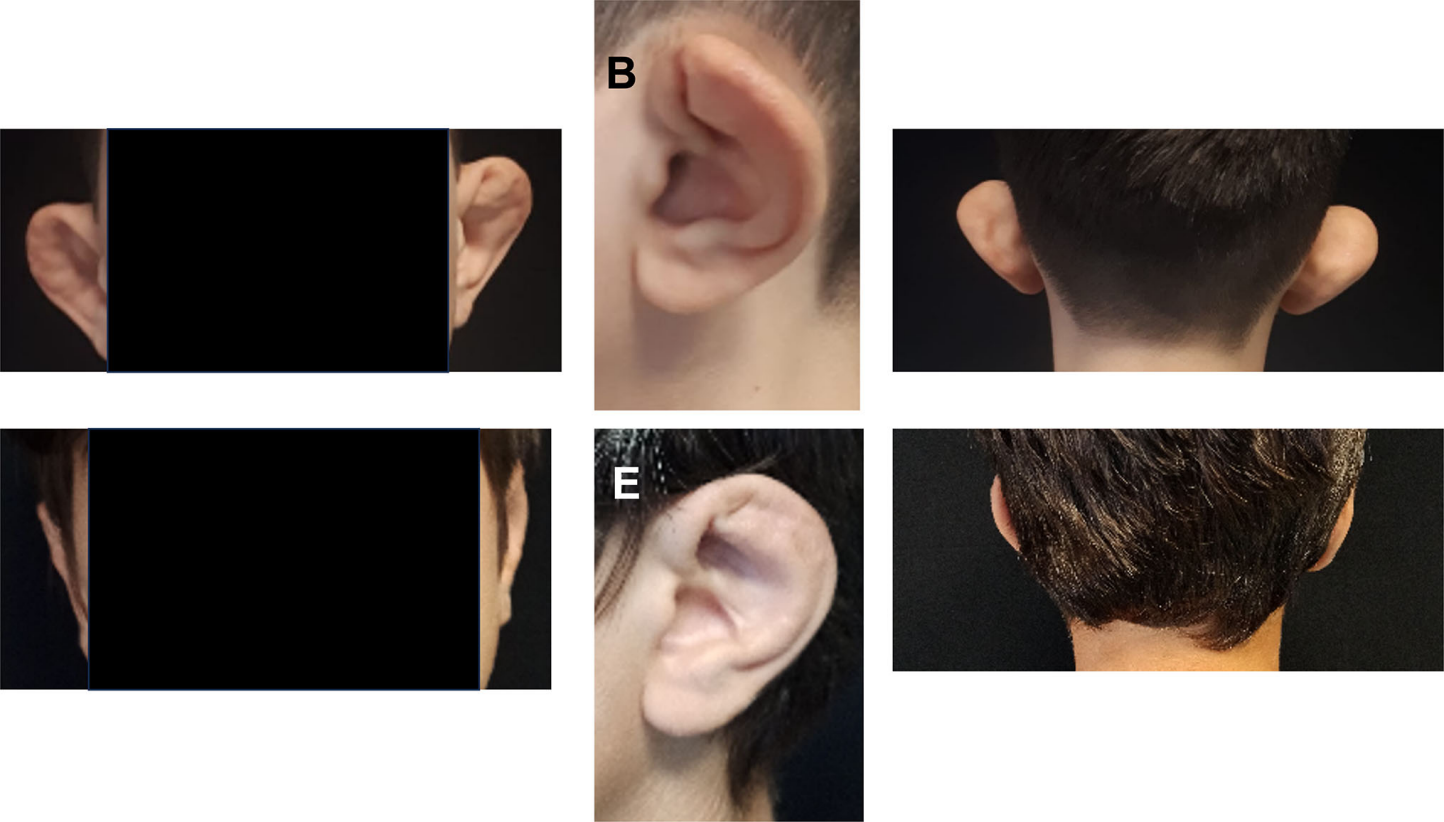
Bilateral case two-year follow-up (patient 1). **A** Preoperative frontal view. **B** Preoperative lateral view. **C** Preoperative posterior view. **D** Post-surgery frontal view. **E** Post-surgery lateral view. **F** Post-surgery posterior view

**Fig. 5:**
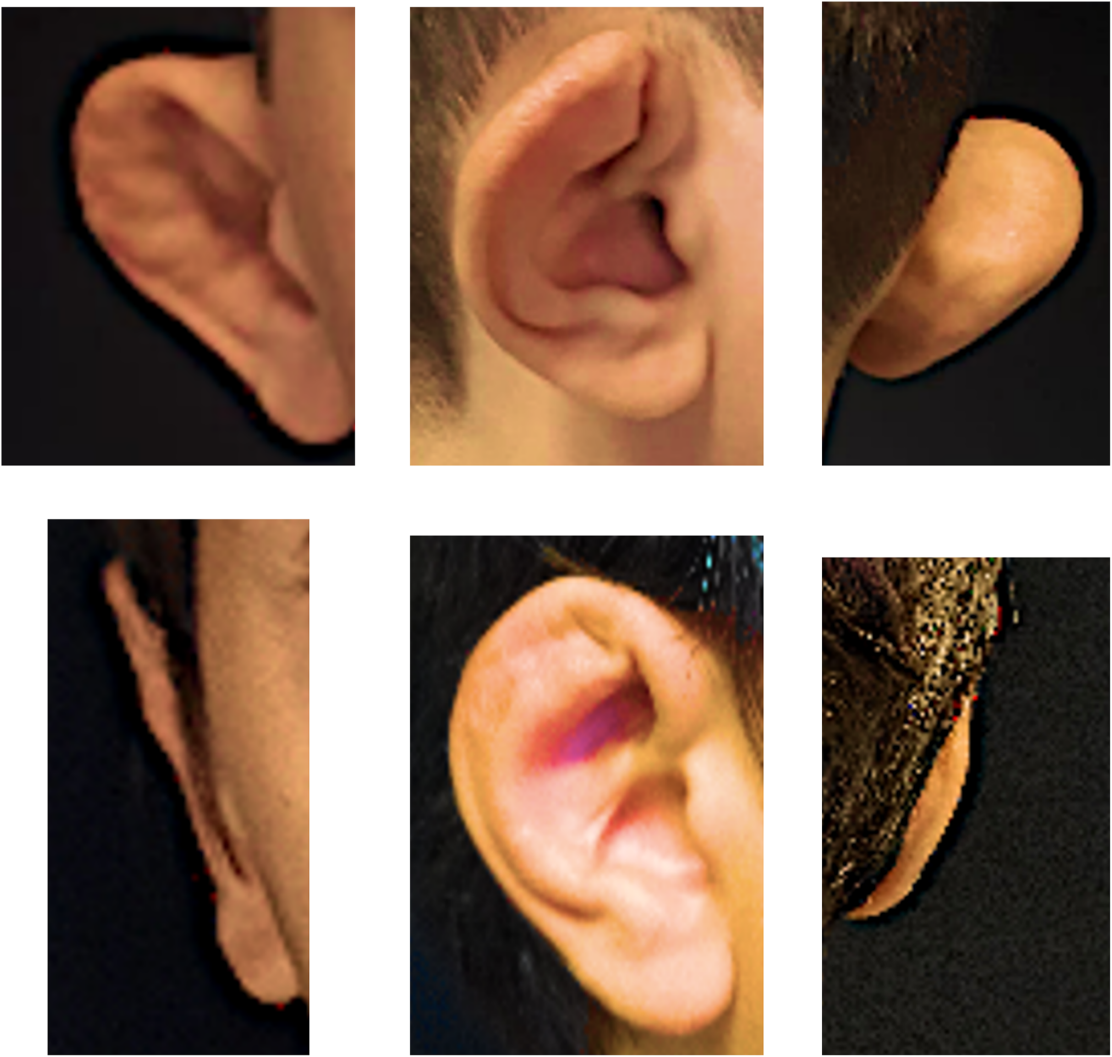
Right side unilateral malformation two-year follow-up (patient 5). **A** Preoperative frontal view. **B** Preoperative lateral view. **C** Preoperative posterior view. **D** Post-surgery frontal view. **E** Post-surgery lateral view. **F** Post-surgery posterior view.

Seven patients underwent the procedure, including 2 bilateral cases (9 ears total). All patients, including bilateral cases, involved ear malformation because of repetitive ear trauma in sports. Three subjects (2 female and a male) had the malformation due to judo practice, 2 males as consequence of boxing, and the other 2 boys from rugby. There were no complications of postoperative bleeding, infection, wound dehiscence, or recurrence of deformity. The mean surgical duration was 52 ± 17 minutes, including the two cases (1 and 2) that involved bilateral surgery, which had durations of 79 and 72 minutes. None of the patients had excessive bleeding during the surgery or in anesthesia recovery; additionally, none of the patients needed reopening of the incision for bleeding or hematoma drainage, there were no instances of infection. The baseline characteristics and outcomes are summarized in Table 1. The follow-up after 24 months showed favorable esthetic outcome in all patients without loss of the shape and/or position of the new antihelix (Figs. 4 and 5). There were no instances of hypertrophic or keloid scar, nor were any patients affected by comorbidities. GCBW showed an average of satisfaction of 99.3 (CI 95%: 93-110; SD: 6.3). The highest satisfaction (average 4; SD 0) was obtained for question 9 (*Has your child's operation affected how well he/she gets on with the rest of the family?*), and the lowest score (average 3.3; SD 0.5) was obtained in question 22 (*Has your child's operation affected how prone he/she is to catch colds or infections?*). No patients needed re-operation.

**Table 1:** Baseline characteristics of patient sample, including severity of malformation and the surgical duration. Severity of malformation is listed by laterality for bilateral cases

Patient	Sex	Site of surgery	Age		Glasgow Children Benefit Questionnaire	Severity of malformation	Surgical duration
1	Male	Bilateral	11	93		5 right, 5 left	79
2	Male	Bilateral	8	94		3 right, 4 left	72
3	Male	Right ear	15	101		1	35
4	Male	Left ear	11	97		3	47
5	Male	Right ear	9	95		2	42
6	Female	Right ear	16	105		2	39
7	Female	Left ear	14	110		3	49

No statistically significant differences were observed in the GCBQ scores between patients who had more severe ear deformation at the baseline compared to the less severe one (p = 0.7). Comparisons between patients who underwent bilateral or monoliteral surgery also showed no significant differences (p = 0.1)

## Discussion

Our use of a modified Valente otoplasty technique to correct cauliflower ear in children allowed for safe, swift, and lasting correction of the deformity. This method restored the esthetic form of the ear with minimal surgical morbidity, as evidenced by higher scores obtained in the GCBW. We attribute the durability of results to combining meticulous debulking of the deformity with helical release and mattress sutures, as described by Valente [8]. The restoration of esthetic form to the ear with a short, single-stage procedure is of particular benefit for children and adolescents who are susceptible to psychologic distress associated with the deformity. In all cases, the surgery was performed in less than an hour per ear. We anticipate that the procedure could be readily performed under local anesthetic or sedation in adults, or in collaborative (adolescent) patients.

As detailed in Table 1, we used this technique to treat different severities of cauliflower malformation [3–7], and in all cases, the results obtained were satisfactory. Great satisfaction was also identified in bilateral surgery (patient 1 and 2 in table 1) and in case of severe deformity before surgery (level 5).

To note that even comparing the different severity of malformation at the baseline and the benefit obtained by using our technique, no statistically different grades of satisfactions were identified. Similar absence of statistically significant difference in satisfaction was observed comparing patients underwent monoliteral or bilateral surgery. These results indicate that our technique could guarantee satisfactory esthetic results independently from the severity of the malformation.

This approach is less aggressive than that proposed by Putri et al., which likely reduces risk of adverse outcomes, and the removal of the cartilage without placement of grafts [5, 6] simplifies care.

None of the children experienced complications or negative outcomes, supporting the safety and low morbidity of this approach. Specifically, the children did not have nor excess of bleeding during the operation, nor needs of reopening the suture due to postoperative bleeding of hematoma. An accurate preparation of the operatory site using 1:20000 adrenaline injected in the correct areas of the ear allows to limit the bleeding during surgery [14]; in particular, the injection both of anterior and posterior ear—the areas that we need to manipulate for our surgery—in addition to avoid bleeding, simplify the dissection of the tissue.

Among the distinctive aspects of this technique are that we dissected both the anterior and the posterior part of the ear, and we removed malformed cartilage from the anterior portion, in contrast to the posterior excision described by Vogelin. During this dissection, we used two different instruments; we dissected the posterior portion of the ear by using monopolar bistoury and the anterior portion by forceps blunt dissection. This approach limited the bleeding in the posterior area of the ear and allowed to adequately dissect the cauliflower area.

Once we removed the anterior malformed portion of the ear by scalpel, we then thinned and softened the remaining cartilage with sharp excision and diamond burr, followed by use of resorbable mattress sutures. Fragilization of the cartilage is necessary to destroy its memory and reduce the risk of recurrence with resorbable sutures, in contrast to the more common use of non-resorbable sutures that can predispose to greater cartilage injury [15] or risk of suture extrusion [15, 16].

Despite the promising preliminary results, the study has several limitations relating to sample size, duration of follow-up, and assessment of outcomes. The technique should be investigated prospectively in a larger and more diverse sample of patients including different ages into adulthood and backgrounds, including health status and sociodemographic characteristics to confirm the results are reproducible and generalizable. This case series involved children, in whom cartilages are more flexible than adults; this could have improved the long-term success with use of resorbable suture and might differ in adults. In addition, a broader range of objective and patient-reported outcomes could be used. Future studies on children and adults affected from different severities of cauliflower malformation are also necessary to understand the implications of this technique.

## Conclusions

There is a paucity of literature on surgical correction of cauliflower ear, and we describe a simple, reliable technique that achieves reliable, long-term outcomes. The technique, which is applicable to any severity of cauliflower malformation, is safe, easy to perform, and durable at 2-year follow-up. Additional studies on larger and more diverse patient samples are needed; however, this technique combining meticulous debulking, helical release, and mattress suturing appears a promising surgical approach for alleviating the morbidity associated with cauliflower ear.

### Supplementary Information

Below is the link to the electronic supplementary material.

Supplementary file1 (MP4 490565 KB)
